# Unraveling Main Limiting Sites of Photosynthesis under Below- and Above-Ground Heat Stress in Cucumber and the Alleviatory Role of Luffa Rootstock

**DOI:** 10.3389/fpls.2016.00746

**Published:** 2016-06-02

**Authors:** Hao Li, Golam J. Ahammed, Guona Zhou, Xiaojian Xia, Jie Zhou, Kai Shi, Jingquan Yu, Yanhong Zhou

**Affiliations:** ^1^Department of Horticulture, Zhejiang UniversityHangzhou, China; ^2^College of Horticulture, Northwest A&F UniversityYangling, China; ^3^Zhejiang Provincial Key Laboratory of Horticultural Plant Integrative BiologyHangzhou, China

**Keywords:** cucumber, grafting, heat stress, HSP70, luffa, oxidative stress, photosynthesis

## Abstract

Photosynthesis is one of the most thermo-sensitive processes in plants. Although the severity of heat stress could be attenuated by grafting approach, the primary damaged site of photosynthesis system under heat stress and the regulatory mechanism of rootstock-mediated heat tolerance are poorly understood. In the current study, cucumber plants grafted onto their own roots and heat-tolerant luffa roots were exposed to root-zone heat (25/40°C) and aerial heat (40/25°C) individually and in combination (40/40°C) to understand the response of photosynthetic process by investigating energy absorption and distribution, electron transport in photosystem (PS) II and I, and CO_2_ assimilation. According to the results, root-zone heat stress inhibited photosynthesis mainly through decreasing Rubisco activity, while aerial heat stress mainly through inhibiting PSII acceptor side. The imbalance in light absorption and utilization resulted in accumulation of reactive oxygen species that caused damage to photosynthetic apparatus, forming a vicious cycle. On the contrary, grafting cucumber onto heat-tolerant luffa rootstock alleviated heat-induced photosynthetic inhibition and oxidative stress by maintaining higher root vitality, HSP70 accumulation, and antioxidant potential.

## Introduction

As sessile organisms, plants perceive various environmental stimuli that often appear as stress such as extreme temperature, drought, and salinity in their natural ecosystems. The episodes of heat, especially extremity and severity of heat events such as heat wave and/or hot days will become more prevalent in the future because of global climate change (IPCC, 2007). Heat stress severely affects the stability of biomembranes, RNA, various proteins, and cytoskeleton structures, and alters the efficiency of enzymatic reactions in the cell, and thus limiting a range of physiological processes (McClung and Davis, 2010; Ruelland and Zachowski, 2010).

Photosynthesis is an important process for energy production, which is particularly sensitive to heat stress (Berry and Björkman, 1980). There are, at least, three major stress-sensitive sites in the photosynthetic machinery such as photosystem II (PSII) with its electron donor (OEC) and acceptor (Q_A_, PQ) (Heckathorn et al., 1998; Pospisl and Tyystjarvi, 1999; Wen et al., 2005), carbon fixation with the key enzymes such as Rubisco and Rubisco activase (Crafts-Brandner and Salvucci, 2000; Salvucci and Crafts-Brandner, 2004; Sharkey, 2005), and thylakoid membrane (Morgan-Kiss et al., 2002; Schrader et al., 2004; Vener, 2007). However, there is still discrepancy in literature concerning the primary targets of heat that cause inhibition in photosynthesis.

As most cellular reactions are coupled to each other, heat-induced disruption of the steady-state flux of metabolites eventually results in accumulation of toxic by-products, such as ROS including 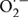 , H_2_O_2_, and ⋅OH. As strong oxidant, ROS could lead to the degradation of chlorophyll and decrease of photochemical efficiency of photosystem II (PSII) (Woo et al., 2004). Moreover, ROS accelerate damage to PSII by damaging the D1 protein which forms a heterodimer with the D2 protein in the reaction center (RC) of PSI (Aro et al., 1993; Andersson and Barber, 1996) and inhibiting the repair of photodamaged PSII through suppressing the *de novo* synthesis of proteins and affecting the stability of thylakoid membrane (Nishiyama et al., 2005, 2006; Sharkey, 2005). To defend against ROS toxicity, plants have developed multiple detoxification systems, including antioxidant enzymatic systems [i.e., SOD, APX, CAT, and glutathione peroxidase (GPX)] and oxidant scavengers [i.e., ascorbate, GSH, and tocopherol], both of which enable the rapid removal of these toxic compounds (Mittler, 2002).

To survive heat stress, plants have evolved a variety of responses to elevated temperatures that minimize damage, ensure proper cellular homeostasis, and enable the organism to function normally. Molecular sensors distributed in different cellular compartments sense increased temperature, generate signals such as Ca^2+^, H_2_O_2_, phosphatidylinositol-4,5-bisphosphate (PIP2), and then activate different transcriptional regulators such as heat shock factor (HSF) via activation of several calcium-dependent protein kinases (CDPKs) and multiple mitogen-activated protein kinases (MAPKs) (Mittler et al., 2012). Finally, protection mechanisms are induced to maintain normal physiological processes including photosynthesis. Heat shock proteins (HSPs), which act as molecular chaperones, are remarkably induced to protect cellular proteins against irreversible heat-induced damage (Boston et al., 1996). Likewise, ROS-scavenging mechanisms are also activated to alleviate heat-induced oxidative stress (Larkindale and Knight, 2002; Suzuki and Mittler, 2006).

Plant photosynthetic capacity is largely dependent on the vitality of roots that maintain the mineral and water states in leaves through proper acquisition of ion and water from root zone area. Importantly, uptake and translocation of water and nutrients, and related physiological and molecular metabolism in leaves are regulated by the root-sourced signals such as abscisic acid (Sauter et al., 2002; Li et al., 2014a). When the roots suffer from stresses such as water and temperature stress, photosynthetic rate is drastically decreased, even goes to below zero depending on the extremity of stress. As a remedy, grafting a sensitive genotype onto tolerant rootstock could substantially alleviate such photosynthetic inhibition in dicotyledonous plants (Zhou et al., 2007; Li et al., 2014b). Nonetheless, to date, how tolerant rootstocks regulate photosynthesis process under stress conditions remains largely underexplored.

Cucumber is one of the important horticultural crops, highly sensitive to heat stress. Previously, we observed that the photosynthetic rate of cucumber was significantly decreased under heat stress; however, such inhibition could be alleviated by using luffa as rootstock (Li et al., 2014a,b). In the present study, we tried to clarify the primary damaged sites of photosynthesis system following heat stress and to understand the regulatory mechanism controlling luffa rootstock-mediated photosynthetic improvement in cucumber. The response of photosynthetic process including energy absorption and distribution, photosynthetic electron transport, CO_2_ assimilation, and root vitality, ROS-generating and -scavenging, and the expression of HSP70 under different aerial and root-zone temperature regimes were investigated. This study adds further evidence concerning primary limiting sites due to heat stress and also provides novel insights into the mechanism of luffa rootstock-mediated heat stress mitigation in cucumber.

## Materials and Methods

### Plant Materials and Treatments

Two different grafted plants, cucumber (*Cucumis sativus* L., cv. Jinyan No. 4, *Cs*) grafted onto cucumber and luffa (*Luffa cylindrical* Roem., cv. Xiangfei No. 236, *Lc*), were used. For rootstocks, seeds of cucumber and luffa were sown directly into trays filled with a mixture of peat/vermiculite (3/1, v/v), and for scion, cucumber seeds were sown 7 days later. When the cotyledon of the cucumber sown for scion expanded, a ‘top approach grafting’ was performed (Davis et al., 2008), and the resulting two groups of seedlings were designated as *Cs/Cs* and *Cs/Lc* according to the rootstock species, respectively. The grafted plants were maintained at a constant humidity of 95–100%, a PPFD of 50 μmol m^-2^ s^-1^ and 25–30°C for 7 days. The seedlings were then transferred to growth chambers with the following environmental conditions: 12-h photoperiod, temperature of 25/18°C (day/night) and PPFD of 600 μmol m^-2^ s^-1^. The plants were watered daily and fertilized with Hoagland’s nutrient solution every 2 days. Upon the appearance of the first true fully expanded leaves, a group of eight seedlings was transplanted into a container (40 cm × 25 cm × 15 cm) filled with Hoagland’s nutrient solution.

At the four-leaf stage, *Cs/Cs* and *Cs/Lc* plants were exposed to root-zone heat (25/40°C, shoot/root temperature), aerial heat (40/25°C), and the combined heat (40/40°C) as described previously (Li et al., 2014a). A 12-h photoperiod and PPFD of 600 μmol m^-2^ s^-1^ were maintained for all treatments. After exposure to heat treatment for 48 h, the CO_2_ assimilation, the chlorophyll fluorescence, and the accumulation of H_2_O_2_ and 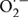 were measured in the third leaf from the bottom. At the same time, leaf and root samples were harvested and stored at -80°C until used for biochemical and gene transcript analysis.

To study the role of HSP70 in luffa-induced heat tolerance, *Cs/Cs* and *Cs/Lc* plants were pretreated with KNK437 and Quercetin (Sigma–Aldrich), inhibitor of HSP70 gene expression, for 12 h before imposition of heat treatment (40/40°C). KNK437 and Quercetin were dissolved in DMSO to prepare a 100 mM stock solution and diluted to 200 μM with Milli-Q water. The corresponding amount of DMSO in the same buffer was used as control. After exposure to a 48 h heat treatment, the chlorophyll fluorescence was measured in the third leaf from the bottom.

### Gas Exchange and Chlorophyll Content Measurements

The gas exchange of attached leaves was measured using an infrared gas analyser, Li-Cor-6400 (Li-Cor Inc., Lincoln, NE, USA), at a temperature of 25°C, a relative humidity of 85%, a cuvette air flow rate of 500 ml min^-1^ and an ambient CO_2_ concentration of 380 μmol mol^-1^. A PPFD of 1000 μmol m^-2^s^-1^ was provided by a mixture of red and blue LEDs.

Leaf chlorophyll (chl a and chl b) were extracted in 80% acetone and their contents were determined according to the method of Arnon (1949).

### Measurement of Quantum Yield and Electron Transport of PSI and PSII

Quantum yield of PSI [Y(I)] and PSII [Y(II)] of grafted plants was measured simultaneously with a Dual-PAM-100 system (Heinz Walz, Germany) on the measure mode of Fluo + P700 (Pfündel et al., 2008). All seedlings were dark-adapted for 30 min prior to measuring *F*_0_, the minimum fluorescence, by a measuring light at low intensity (<0.1 μmol m^-2^ s^-1^). A saturating pulse (10,000 μmol photons m^-2^ s^-1^) was then applied to detect *F*_m_ (the maximum fluorescence after dark adaptation). The maximal change of P700 signal (*P*_m_) was measured through application of a saturation pulse (10,000 μmol photons m^-2^ s^-1^) after illumination of far-red light for 10 s. A saturating pulse with duration of 300 ms was applied every 20 s after the onset of the actinic light to determine the maximum fluorescence signal (*F*_m′_) and maximum P700+ signal (*P*_m′_) under the actinic light (27 μmol photons m^-2^ s^-1^). The slow induction curve was recorded for 300 s to achieve the steady state of the photosynthetic apparatus, and then the actinic light was turned off. Data [Y(II), ETR(II), Y(NPQ), Y(NO), Y(I), ETR(I), Y(NA), Y(ND), qP] derived after the final saturating pulse (**Table 1**), were recorded and used for analysis of activities of PSI and PSII. According to Pfündel et al. (2008): Y(I) = (*P*_m′_-*P*)/*P*_m_, Y(NA) = (*P*_m_-*P*_m′_)/*P*_m_, Y(ND) = (*P-P*_o_)/*P*_m_, ETR(I) = 0.5×Y(I)×PAR×0.84μmol×m^-2^×s^-1^. According to Kramer et al. (2004): Y(II) = (*F*_m′_-*F*)/*F*_m′_, ETR(II) = 0.5×Y(II)×PAR×0.84 μmol×m^-2^×s^-1^, qP = (*F*_m′_/-*F*)/(*F*_m′_-*F*_o′_), qL = qP(*F*_o′_/*F*′), NPQ = (*F*_m_-*F*_m′_)/*F*_m′_, Y(NO) = 1/NPQ + 1 + qL ((*F*_m_/Fo)-1), Y(NPQ) = 1-Y(II)-Y(NO).

**Table 1:** Chlorophyll content and chlorophyll fluorescence parameters in cucumber plants as influenced by rootstock and root-zone or/and aerial heat stress.

Temperature (°C)	25/25		25/40		40/25		40/40	
				
	*Cs/Cs*	*Cs/Lc*	*Cs/Cs*	*Cs/Lc*	*Cs/Cs*	*Cs/Lc*	*Cs/Cs*	*Cs/Lc*
Chl (mg g^-1^ FW)	1.40 ± 0.07^a^	1.41 ± 0.02^a^	1.42 ± 0.09^a^	1.37 ± 0.15^a^	1.50 ± 0.15^a^	1.54 ± 0.14^a^	1.46 ± 0.16^a^	1.44 ± 0.12^a^
ABS/CSm	1.17 ± 0.11^a^	1.20 ± 0.14^a^	1.10 ± 0.09^a^	1.13 ± 0.15^a^	1.18 ± 0.12^a^	1.17 ± 0.07^a^	1.10 ± 0.09^a^	1.11 ± 0.08^a^
TR/CSm	0.99 ± 0.08^a^	1.06 ± 0.04^a^	0.73 ± 0.07^c^	0.79 ± 0.09^bc^	0.62 ± 0.06^d^	0.85 ± 0.04^b^	0.43 ± 0.03^e^	0.61 ± 0.01^d^
ET/CSm	0.54 ± 0.02^a^	0.57 ± 0.04^a^	0.36 ± 0.02^c^	0.44 ± 0.04^b^	0.27 ± 0.02^d^	0.42 ± 0.04^b^	0.20 ± 0.04^e^	0.32 ± 0.06^cd^
φ_P0_	0.76 ± 0.01^ab^	0.77 ± 0.03^a^	0.64 ± 0.06^d^	0.72 ± 0.03^bc^	0.55 ± 0.03^e^	0.67 ± 0.04^cd^	0.43 ± 0.06^f^	0.58 ± 0.05^e^
φ_E0_	0.43 ± 0.03^a^	0.45 ± 0.02^a^	0.31 ± 0.03^cd^	0.38 ± 0.02^b^	0.24 ± 0.02^e^	0.34 ± 0.01^c^	0.19 ± 0.03^f^	0.28 ± 0.02^d^
Y(ll)	0.43 ± 0.04^a^	0.44 ± 0.03^a^	0.32 ± 0.01^c^	0.36 ± 0.01^b^	0.21 ± 0.02^d^	0.32 ± 0.02^c^	0.14 ± 0.02^e^	0.22 ± 0.03^d^
ETR(II)	49.9 ± 4.29^a^	50.9 ± 3.84^a^	36.9 ± 1.68^c^	42.2 ± 1.57^b^	24.6 ± 2.27^d^	36.9 ± 2.68^c^	15.9 ± 2.82^e^	25.4 ± 3.06^d^
Y(l)	0.55 ± 0.04^a^	0.54 ± 0.02^a^	0.44 ± 0.00^bc^	0.47 ± 0.03^b^	0.38 ± 0.01^cd^	0.44 ± 0.05^bc^	0.33 ± 0.02^d^	0.42 ± 0.04^bc^
ETR(I)	68.8 ± 4.35^a^	62.8 ± 2.28^a^	50.4 ± 0.56^bc^	55.0 ± 3.15^b^	43.9 ± 1.35^cd^	50.7 ± 6.12^cd^	37.7 ± 6.05^d^	48.8 ± 4.37^cd^
Y(NPQ)	0.30 ± 0.07^ab^	0.25 ± 0.03^b^	0.30 ± 0.01^ab^	0.33 ± 0.05^ab^	0.31 ± 0.06^ab^	0.37 ± 0.08^a^	0.24 ± 0.04^b^	0.33 ± 0.03^ab^
Y(ND)	0.37 ± 0.03^c^	0.38 ± 0.03^bc^	0.50 ± 0.02^a^	0.46 ± 0.02^a^	0.44 ± 0.06^ab^	0.48 ± 0.03^a^	0.38 ± 0.03^bc^	0.44 ± 0.01^ab^
Y(NO)	0.27 ± 0.04^e^	0.31 ± 0.05^de^	0.38 ± 0.00^cd^	0.31 ± 0.05^de^	0.47 ± 0.06^b^	0.31 ± 0.07^de^	0.62 ± 0.03^a^	0.45 ± 0.06^bc^
Y(NA)	0.08 ± 0.01^cd^	0.08 ± 0.01^cd^	0.07 ± 0.02^cd^	0.06 ± 0.02^d^	0.18 ± 0.05^b^	0.09 ± 0.02^cd^	0.29 ± 0.05^a^	0.14 ± 0.04^bc^


*Data are the means ± SD of four replicates. Means denoted by the same letter did not differ significantly according to Tukey’s test (*P* < 0.05).*

### Chlorophyll Fluorescence Kinetics and Calculation of JIP-Test Parameters

Chl a fluorescence transients were measured with a Dual-PAM-100 system (Heinz Walz, Germany). All samples were adapted to the dark for 15 min. The chlorophyll fluorescence transients were recorded up to 1 s on a logarithmic time-scale. Data were acquired every 20 μs. The polyphasic fluorescence induction kinetics was analyzed according to the JIP test that reflected valuable information on photosystem II (PSII) function (Strasser and Govindjee, 1992). In the present study, the following data were directly obtained from the fast-rise kinetic curves: *F*_0_ (initial fluorescence) was measured at 20 μs, when all PSII RCs are open; *F*_300__μs_ is the fluorescence at 300 μs; *F*_j_ and *F*_i_ are the fluorescence intensity at step J (2 ms) and at step I (30 ms), respectively; *F*_m_ (maximal fluorescence) is the peak of fluorescence at the step P when all RCs are closed; Area is total complementary area between fluorescence induction curve. Selected parameters quantifying PSII behavior were calculated from the original data following the formulae as shown in **Supplementary Table S1** (Strasser and Strasser, 1995; Strasser et al., 2004).

### Rubisco Activity Determination

The frozen leaf samples were homogenized using a chilled pestle and mortar with cooled extraction buffer containing 50 mM Tris-HCl (pH 7.5), 1 mM ethylenediaminetetraacetic acid (EDTA), 1 mM MgCl_2_, 12.5% (v/v) glycerin, 10% polyvinylpyrrolidone (PVP), and 10 mM β-mercaptoethanol. The homogenates were centrifuged at 15,000 *g* for 15 min at 4°C.

Rubisco activity was measured spectrophotometrically by coupling 3-phosphoglyceric acid formation with NADH oxidation at 25°C according to Lilley and Walker (1974) with some modifications. The total activity was assayed following enzyme activation, which was achieved by pre-incubating for 15 min in a 0.1 ml mixture containing 33 mM Tris-HCl (pH 7.5), 0.67 mM EDTA, 33 mM MgCl_2_, and 10 mM NaHCO_3_. The initial and total Rubisco activity measurements were then carried out in a 0.1 ml reaction medium containing 5 mM HEPES-NaOH (pH 8.0), 1 mM NaHCO_3_, 2 mM MgCl_2_, 0.25 mM dithiothreitol (DTT), 0.1 mM EDTA, 1 U creatine phosphokinase, 1 U 3-phosphoglyceric phosphokinase, 1 U glyceraldehyde 3-phosphate dehydrogenase, 0.5 mM ATP, 0.015 mM NADH_2_, 0.5 mM phosphocreatine, 0.06 mM RuBP, and 10 μl of extract. The change in absorbance at 340 nm was monitored for 90 s.

### Determination of NADPH and NADP^+^

The fresh leaf samples (0.3 g) were homogenized using a chilled pestle and mortar with 3.0 ml of 0.2 M HCI for NADPH determination or 3.0 ml of 0.2 M NaOH for NADP^+^ determination. Each homogenate was heated in a boiling water bath for 5 min, cooled in an ice bath, then centrifuged at 10,000 *g* at 4°C for 10 min. Supernatants were neutralized with respectively 0.2 M NaOH or HCI and centrifuged at 10,000 *g* at 4°C for 10 min. Final supernatants were transferred to separate tubes and kept on ice for coenzyme assay.

Enzyme cycling assays of NADP(H) was performed in low light with MTT as the terminal electron acceptor according to the method described by Zhao et al. (1987) with some modifications. Briefly, 50 μl of 4 μM NADPH solutions or sample supernatants were added to 500 μl mixture containing 0.1 M Tricine–NaOH buffer (PH 8.0), 10 mM EDTA(disodium salt), 1 mM MTT, 2 mM phenazineethosulfate (PES), 5 mM G6P and incubated for 5 min at 37°C. Enzyme cycling was initiated by adding 2 U G6PDH solution and 40 min later was stop by adding 500 μl of 6 M NaCl. With each biological sample, a blank measurement was also made by adding 0.1 M Tricine-NaOH buffer instead of enzyme. After centrifuged at 10,000 *g* at 4°C for 5 min, the supernatants were carefully removed and the pellet was solubilized in 1 ml 96% ethanol. Finally, absorbance at 570 nm was determined.

### Measurement of Root Vitality and Leaf Water Potential

Root vitality was determined by triphenyltetrazolium chloride (TTC) method according to Clemensson-lindell (1994). The fresh roots were cut into small pieces 1-to 2-mm long, and 0.2 g of them were incubated with 6 ml of 0.6% (w/v) TTC in 0.06 M Na_2_HPO_4_–KH_2_PO_4_ at 37°C for 3 h and 0.05% (v/v) Tween 20 were added and the samples were vacuum-infiltered for 15 min. After incubation, the root pieces were washed twice with 5 ml of distilled water. Then, the samples were extracted in 95% (v/v) ethanol at 80°C for 15 min. Absorption at 520 nm was measured by Multimode Plate Reader Label-free System (PerkinElmer, MA, USA).

Leaf water potential (Ψ_leaf_) was determined on intact excised leaves from plants with a dew point potential meter (WP4-C, Decagon Devices, Inc., Pullman, WA, USA).

### Detection of 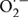 and H_2_O_2_ Generation

The 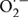 accumulation was visualized by nitroblue tetrazolium (NBT) staining. Leaf disks (1.5 cm in diameter) were directly infiltrated with 0.1 mg ml^-1^ NBT in 25 mM K-HEPES buffer (pH7.8) and incubated at 25°C in the dark for 4 h. Then, the leaf disks were rinsed in 95% (v/v) ethanol for 10 min at 95°C and photographed with a digital camera (Canon EOS 5D; Canon Inc., Tokyo, Japan).

For the histochemical staining of H_2_O_2_, leaves were detached and placed in a solution containing 1 mg ml^-1^ 3,3′-diaminobenzidine (DAB, pH 5.5) for 6 h after a light vacuum infiltration. Leaf disks were boiled in 95% (v/v) ethanol for 10 min, stored in 50% glycerol, and then photographed with a digital camera (Canon EOS 5D; Canon Inc., Tokyo, Japan) or an Olympus motorized system microscope (BX61, Olympus Co., Tokyo, Japan) at 400 magnifications (Thordal-Christensen et al., 1997).

### Assay of Glutathione and Antioxidant Enzymes

For the measurement of GSH and GSSG, plant leaf tissue (0.3 g) was homogenized in 2 mL of 5% metaphosphoric acid containing 2 mM EDTA and centrifuged at 4°C for 15 min at 12,000 *g*. GSH and oxidized glutathione (GSSG) contents were determined according to Rao et al. (1995) by an enzymatic recycling method.

Antioxidant enzyme activities in leaves were assayed with spectrophotometric methods. The protein content was determined according to the Bradford and Williams (1976) method. The SOD activity was determined with the Stewart and Bewley (1980) method based on the photochemical reduction of NBT. The CAT activity was measured by monitoring the rate of H_2_O_2_ decomposition at A240 according to Cakmak and Marschner (1992). The GR activity was measured according to Foyer and Halliwell (1976) which was based on the rate of decrease in the absorbance of NADPH at 340 nm. The ascorbate peroxide (APX) activity was measured by monitoring the rate of ascorbate oxidation at 290 nm according to Nakano and Asada (1981).

### Western Blotting for HSP70

Heat Shock Protein 70 was extracted as described by Zhang and Klessig (1997). Briefly, 0.3 g of the frozen leaf was ground in liquid nitrogen in 0.6 mL extraction buffer [100 mM HEPES, pH 7.5, 5 mM EDTA, 5 mM ethylene glycol tetraacetic acid (EGTA), 10 mM DTT, 10 mM Na_3_VO_4_, 10 mM NaF, 50 mM b-glycerophosphate, 1 mM phenylmethylsulfonyl fluoride, 5 mg mL^-1^ antipain, 5 mg mL^-1^ aprotinin, 5 mg mL^-1^ leupeptin, 10% glycerol and 7.5% polyvinylpolypyrrolidone (PVP)]. After centrifugation at 13,000 *g* for 20 min, the supernatants were transferred into clean tubes. The protein concentrations of the extracts were determined using the Bio-Rad protein assay kit using bovine serum albumin (BSA) as the standard. Denatured protein extracts were separated by 12.5% sodium dodecyl sulfate–polyacrylamide gel electrophoresis (SDS–PAGE), and the proteins were then transferred to a nitrocellulose membrane (Bio-Rad) by semidry electroblotting. The membrane was blocked for 2 h in TBS buffer (20 mM Tris, pH 7.5, 150 mM NaCl, 0.1% Tween 20, 0.1 mM Na_3_VO_4_) with 5% BSA at room temperature and then incubated for 1 h in TBS buffer (with BSA) containing the Rabbit polyclonal antibody HSP70 (Agrisera, vännäs, Sweden). After incubation with HRP (horseradish peroxidase)-linked antibody (Cell Signaling Technology, Beverly, MA, USA), the complexes on the blot were visualized using an enhanced chemiluminescence kit (Perkin Elmer) following the manufacturer’s instructions. The quantitation of HSP70 was performed by ImageJ.

### RNA Extraction and Quantitative Real-Time PCR (qRT-PCR) Analysis

Total RNA was extracted from grafted cucumber leaves using an RNA extraction kit (Axgen, Union City, CA, USA) according to the supplier’s instructions. DNA contamination was removed using a purifying column. One microgram of total RNA was reverse-transcribed using the ReverTra Ace qPCR RT Kit (Toyobo, Osaka, Japan) following the supplier’s recommendations. The gene-specific primers for qRT-PCR were designed based on their cDNA sequences, as follows: *RBCL* (F, 5′-ATTTGCGAATCCCTACT-3′; R, 3′-AAACCGCTCTACCATAA-5′), *RBCS* (F, 5′-ACCACAGGTCACCAGGAT-3′; R, 3′-GGGCTTGTAGGCGATG-5′), *RCA* (F, 5′-AAGTGAGAAAGTGGGCTGTA-3′; R, 3′-TTGTCATCTTCGGTTGGT-5′), and the actin gene (F, 5′-TGGACTCTGGTGATGGTGTTA-3′; R, 3′-CAATGAGGGATGGCTGGAAAA-5′) was used as an internal control. The qRT-PCR assays were performed using an iCycleriQTM Real-time PCR Detection System (Bio-Rad, Hercules, CA, USA). PCRs were performed using the SYBR Green PCR Master Mix (Takara, Tokyo, Japan). The PCR conditions consisted of denaturation at 95°C for 3 min, followed by 40 cycles of denaturation at 95°C for 30 s, annealing at 58°C for 30 s and extension at 72°C for 30 s. The quantification of mRNA levels was based on the method of Livak and Schmittgen (2001).

### Statistical Analysis

The experiment was a completely randomized design with four replications. Each replicate contained at least 10 plants. Two-way analysis of variance (ANOVA) was used to test for significance, and significant differences (*P* < 0.05) between treatments were determined using Tukey’s test (**Table 2**).

**Table 2:** Statistical information with the *F* and *P*-values of the main factors and interactions.

	Temperature	Rootstock	Interaction
	(*F*/*P* Value)	(*F*/*P* Value)	(*F*/*P* Value)
*Asa*f (**Figure 1**)	104.47/<0.001	57.75/<0.001	5.40/0.006
Pl_ABS_ (**Figure 1**)	123.40/<0.001	56.54/<0.001	10.17/<0.001
OEC (**Figure 2A**)	18.43/<0.001	21.32/<0.001	4.73/<0.010
RC/CS_m_ (**Figure 2A**)	80.63/<0.001	39.20/<0.001	6.13/<0.003
qP (**Figure 2A**)	86.66/<0.001	32.57/0.001	5.97/<0.006
S_m_ (**Figure 2A**)	51.75/<0.001	20.65/<0.001	1.83/<0.170
NADP^+^+ NADPH (**Figure 2B**)	0.25/0.858	4.51/0.050	1.08/0.387
NADP^+^ (**Figure 2B**)	4.16/<0.023	7.09<0.017	2.17/0.131
NADPH (**Figure 2B**)	10.24/0.001	0.04<0.839	1.20/0.342
NADP^+^/NADPH (**Figure 2B**)	18.03<0.001	3.66<0.074	3.52/<0.039
*RCBL* (**Figure 3A**)	10.28/0.001	105.64/<0.001	16.35/<0.001
*RCBS* (**Figure 3A**)	139.21/<0.001	598.50/<0.001	136.33/0.001
*RCA* (**Figure 3A**)	38.32/<0.001	127.18/<0.001	28.83/<0.001
Total Rubisco (**Figure 3B**)	4.22/0.022	5.81/0.028	1.71/0.205
Initial Rubisco (**Figure 3B**)	54.36/<0.001	41.86/0.001	6.23/0.005
Rubisco activation rate (**Figure 3B**)	48.23/<0.001	25.58/<0.001	2.15/0.133
GSH (**Figure 5B**)	44.14/0.001	13.10/<0.002	1.44/<0.268
GSSG (**Figure 5B**)	7.49/0.002	21.23/<0.001	4.29/0.021
GSH/GSSG (**Figure 5B**)	32.37/<0.001	62.56/<0.001	4.05/0.026
REC (%) (**Figure 5B**)	47.30/<0.001	33.16/0.001	3.315/0.078
SOD (**Figure 5B**)	30.43/<0.001	42.75/<0.001	4.52/0.018
CAT (**Figure 5B**)	106.40/<0.001	24.98/<0.001	4.80/0.014
GR (**Figure 5B**)	81.76/<0.001	31.95/<0.001	3.35/0.045
APX (**Figure 5B**)	29.71/<0.001	11.68/0.004	3.116/0.056
Chl (**Table 1**)	1.19/0.346	0.018/0.895	0.15/0.929
ABS/CSm (**Table 1**)	1.179/0.339	0.158/0.694	0.053/0.983
TR/CSm (**Table 1**)	103.42/<0.001	45.21/0.001	4.448/0.013
ET/CSm (**Table 1**)	97.27/<0.001	57.534/<0.001	4.24/0.015
φ_P0_ (**Table 1**)	53.15/<0.001	37.27/<0.001	3.75/0.024
φ_E0_ (**Table 1**)	106.84/<0.001	69.67/0.001	5.12/0.007
Y(II) (**Table 1**)	113.18/<0.001	35.12/<0.001	4.28/0.021
ETR(II) (**Table 1**)	113.11/0.001	35.11/<0.001	4.23/0.022
Y(I) (**Table 1**)	36.56/<0.001	14.00/0.002	3.10/0.056
ETR(I) (**Table 1**)	37.32/<0.001	14.40/0.002	3.16/0.054
Y(NPQ) (**Table 1**)	2.02/0.152	2.39/0.141	2.22/0.125
Y(ND) (**Table 1**)	12.51/<0.001	1.69/0.212	1.98/0.158
Y(NO) (**Table 1**)	28.27/0.001	20.91/0.001	6.06/0.006
Y(NA) (**Table 1**)	24.57/<0.001	22.59/<0.001	7.38/0.003
*Ci* (**Figure 4**)	26.87/<0.001	11.85/0.002	5.54/0.005
*Gs* (**Figure 4**)	362.81/<0.001	1.86/0.186	5.33/0.006
Ψ_leaf_ (**Figure 4**)	137.95/<0.001	118.52/0.001	51.06/0.001
Root activity (**Figure 4**)	43.63/0.001	41.30/<0.001	4.31/0.021


## Results

### Photosynthetic Responses of Grafted Cucumber Plants to Heat Stress

**FIGURE 1:**
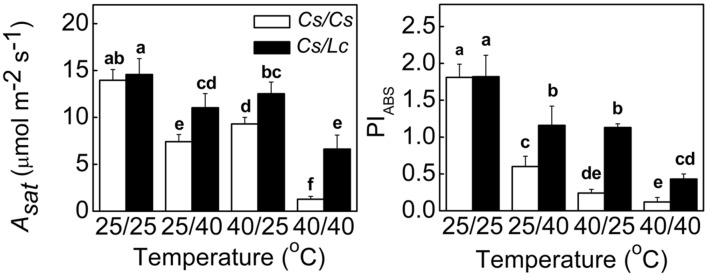
**Changes in the *A_sat_* and photosynthetic performance (PI_ABS_) in cucumber plants as influenced by rootstock and root-zone or/and aerial heat stress.** All data were determined at 48 h after heat treatment. The bars (means ± SD, *n* = 4) labeled with different letters are significantly different at *P* < 0.05 according to Tukey’s test.

The *A_sat_* and PI_ABS_ are useful indicators of photosynthetic performance. As shown in **Figure 1**, root-zone heat (25/40°C) and aerial heat (40/25°C) individually and in combination (40/40°C, shoot/root) significantly decreased the *A_sat_* and PI_ABS_ in self-grafted (*Cs/Cs*) cucumber plants. Most pronounced decrease was observed in the 40/40°C treatment, followed by 25/40°C and 40/25°C. Significantly, the leaves of reciprocally grafted cucumber (*Cs/Lc*) plants where luffa was used as rootstock showed much higher *A_sat_* and PI_ABS_ values under heat stress compared to the *Cs/Cs* plants. For example, heat stress decreased the *A_sat_* by 47.0, 33.4, and 90.9%, and PI_ABS_ by 66.9, 86.7, and 93.4% following exposure of *Cs/Cs* plants to 25/40, 40/25, and 40/40°C for 48 h, respectively. On the other hand, decreases in the *Cs/Lc* plants for the *A_sat_* were only 24.4%, 14.1% and 54.5%, while for PI_ABS_ were 36.3, 37.9, and 76.4%, respectively under the same treatments.

### Changes in Photochemical Reactions as Influenced by Heat Stress in Grafted Plants

To explore the reason of drastic reduction in *A_sat_* and PI_ABS_ following heat stress, we firstly analyzed the light energy absorption in *Cs/Cs* and *Cs/Lc* plants. Unlike *A_sat_* and PI_ABS_, the ABS/CS_m_ and chlorophyll contents in *Cs/Cs* and *Cs/Lc* plants remained almost unchanged following 25/40°C, 40/25°C, and 40/40°C temperature regimes (**Table 1**). It is well known that part of the absorbed energy is used to drive photosynthesis (photochemistry). However, the flux channelled to the RC reducing Q_A_ to Q_A_^-^ (called ‘trapping flux’ TR) and the flux transported beyond Q_A_^-^ which is re-oxidized to Q_A_ (called ‘electron transport flux’ ET) were significantly decreased by root-zone or/and aerial heat stress in both grafted plants, resulting in the decreases of φ_P0_ and φ_E0_. Lower TR/CS_m_, ET/CS_m_, φ_P0_, φ_E0_, Y(II), ETRII, Y(I), and ETR(I) were observed in *Cs/Cs* plants after exposure to aerial heat than those in *Cs/Cs* plants after exposure to root-zone heat. *Lc* as rootstock significantly alleviated the decreases in most of these parameters. For example, following 25/40°C, 40/25°C and 40/40°C treatments, ET/CS_m_ and Y(II) in *Cs/Cs* plants were decreased by 33.3, 50.0, 63.0 and 25.6, 51.2%, 67.4, respectively, while those in *Cs/Lc* plants were decreased by 22.8, 26.3, 43.9 and 18.2, 27.3, 50.0%, respectively.

The Y(NPQ) in PSII and Y(ND) in PSI represent heat dissipation related to light protection capacity of plants. After all three heat treatments, Y(NPQ) in both grafted plants remained almost unchanged, while Y(ND) was increased significantly, and relatively greater increase was noticed in *Cs/Lc* plants. Furthermore, Y(NO) in PSII and Y(NA) in PSI represent the extent of optical damage (Kramer et al., 2004). Both Y(NO) and Y(NA) in *Cs/Cs* plants were significantly induced by aerial or/and root-zone heat [except Y(NA) by root-zone heat]. However, *Lc* as rootstock inhibited such increases in Y(NO) and Y(NA). Over all, heat stress caused minor damage and consequently lower decrease in electron transport of PSI than those of PSII due to the higher light protection capacity of PSI to a certain degree (**Table 1**). Moreover, the protection of PSII and PSI by *Lc* rootstock was partially associated with *Lc*-induced light protection capacity.

### Response of the Donor and Acceptor Side in Electron Transport Chain

**FIGURE 2:**
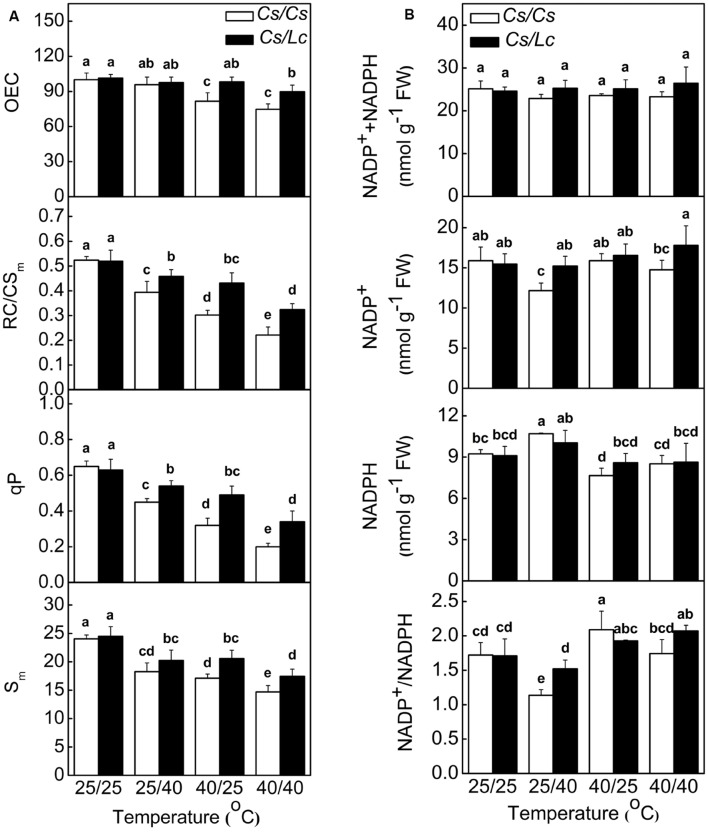
**Changes in PSII components **(A)** and NADP(H) content and redox status **(B)** in cucumber plants as influenced by rootstock and root-zone or/and aerial heat stress.** Samples were harvested at 48 h after heat treatment. The bars (means ± SD, *n* = 4) labeled with different letters are significantly different at *P* < 0.05 according to Tukey’s test.

Oxygen-evolving complex is the primary electron donor, RC/CS_m_ represents the reactive center per cross section, qP (photochemical quenching factor) is a proxy for the redox state of Q_A_ which is the primary electron acceptor after PSII, S_m_ reflects the size of PQ pools, and oxidized nicotin amide adenine (NADP^+^) is the terminal electron acceptor (Farquhar et al., 1980; Strasser et al., 2004). Like ET/CS_m_ and Y(II), OEC, RC/CS_m_, qP, and S_m_ in *Cs/Cs* plants were significantly decreased after heat stress, while root-zone heat caused drastic decreases than aerial heat (**Figure 2A**). However, *Lc* as rootstock alleviated heat-induced decrease of these parameters. NADP^+^ was significantly decreased in *Cs/Cs* plants, while the reduced nicotinamide adenine (NADPH) was increased by root-zone heat (**Figure 2B**). However, the sum of NADPH + NADP^+^ was not altered by root zone or/and aerial heat treatments. Accordingly, the ratio of NADP^+^/NADPH was decreased significantly in *Cs/Cs* plants after root-zone heat stress. *Lc* as rootstock reversed the decrease in NADP^+^ content and ratio of NADP^+^/NADPH under root zone heat stress. Notably, the content of NADP^+^ or/and NADPH and the ratio of NADP^+^/NADPH were almost unchanged after 40/25°C and 40/40°C heat treatments in both *Cs/Cs* and *Cs/Lc* plants.

### Response of Rubisco Activity and Intercellular CO_2_ Concentration to Heat Stress in Grafted Plants

**FIGURE 3:**
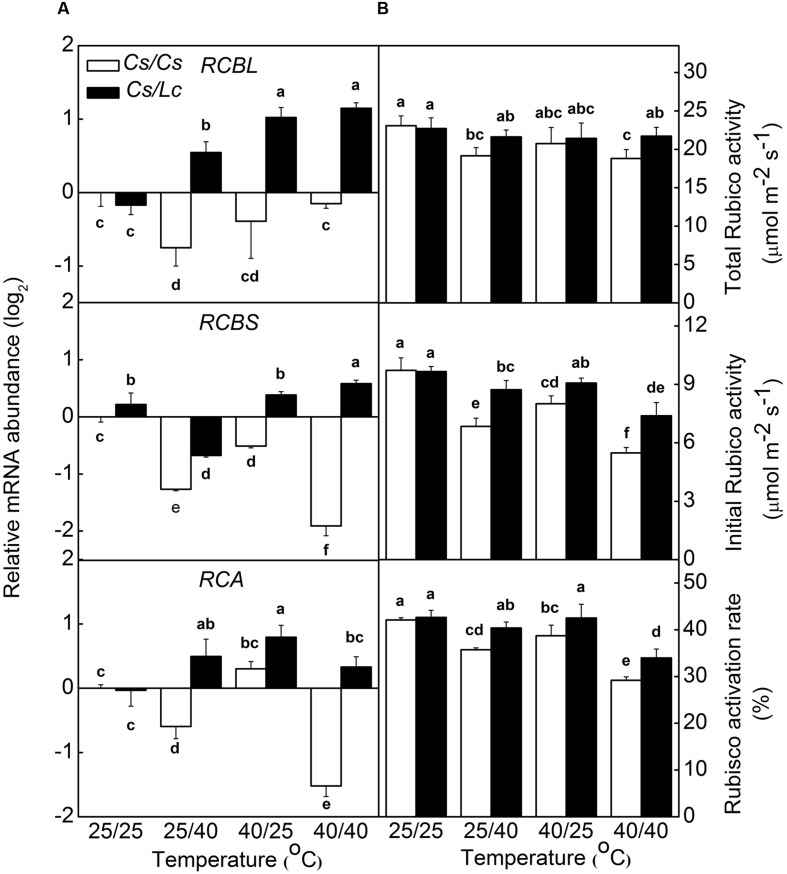
**The steady-state transcript levels for *RCBL*, *RCBS*, *RCA***(A)** and the activities and activation rate of Rubisco **(B)** in response to rootstock and root-zone or/and aerial heat stress.** Samples were harvested at 48 h after heat treatment. The bars (means ± SD, *n* = 4) labeled with different letters are significantly different at *P* < 0.05 according to Tukey’s test.

The regeneration rate of NADP^+^ is partly dependent on the rate of Calvin–Benson Cycle. Therefore, we analyzed the activity and transcript levels of Rubisco, a key rate-limiting enzyme in Calvin-Benson Cycle. At the optimal growth temperature, i.e., 25/25°C, luffa rootstock didn’t affect *RBCL* and *RCA* expression significantly (**Figure 3**). However, heat stress upregulated *RBCL*, *RBCS*, and *RCA* expression in *Cs/Lc* plants, except for *RCBS* in 25/40°C and *RCA* in 25/40°C and 40/40°C treatments. Notably, *Cs/Lc* plants showed relatively higher *RBCL*, *RBCS*, and *RCA* transcripts abundance than *Cs/Cs* plants after 25/40°C, 40/25°C, and 40/40 °C heat treatments for 48 h. Total and initial Rubisco activities and Rubisco activation rate in *Cs/Cs* plants were decreased significantly after exposure to heat stress. However, luffa rootstock alleviated heat stress-induced decreases of Rubisco activities and activation rate. Ultimately, total and initial Rubisco activities and Rubisco activation rate in *Cs/Lc* plants were higher than those in *Cs/Cs* plants after exposure to root-zone or/and aerial heat.

**FIGURE 4:**
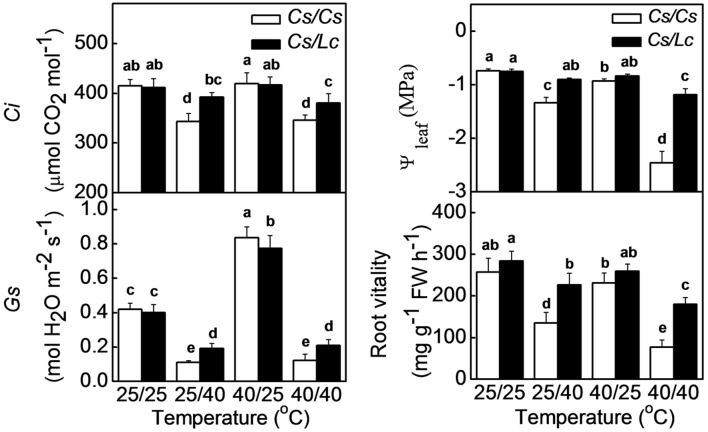
**Intercellular CO_2_, stomatal conductance (*Gs*) and water potential of leaf in response to rootstock and root-zone or/and aerial heat stress and root vitality of cucumber or luffa rootstock as influenced by root-zone or/and aerial heat stress.** All data were determined at 48 h after heat treatment. The bars (means ± SD, *n* = 4) labeled with different letters are significantly different at *P* < 0.05 according to Tukey’s test.

Intercellular CO_2_ concentration is an important factor that impacts Rubisco activity and photosynthetic rate. *Ci* in *Cs/Cs* plants was not affected by aerial heat, but was significantly decreased by root-zone heat and combined heat treatments (**Figure 4**). *Ci* is dependent on the balance between the CO_2_ assimilation and CO_2_ intake through stomata. Similarly, *Gs* was significantly decreased by root-zone as well as combined heat. As stomatal closure is closely associated with the leaf water potential (Ψ_leaf_) which is basically affected by water supply from root, we further analyzed changes in Ψ_leaf_ and root vitality. Ψ_leaf_ and root vitality showed similar change trends as like as *Gs* and *Ci*. However, root-zone heat- and combined heat-induced decreases in *Ci*, *Gs*, Ψ_leaf_, and root vitality were alleviated when cucumber was grafted onto *Lc* rootstock. In both grafted plants, *Ci*, Ψ_leaf_, and root vitality were almost unchanged, but *Gs* was significantly increased after exposure to aerial heat.

### Response of ROS Generation and Scavenging to Heat Stress in Grafted Plants

**FIGURE 5:**
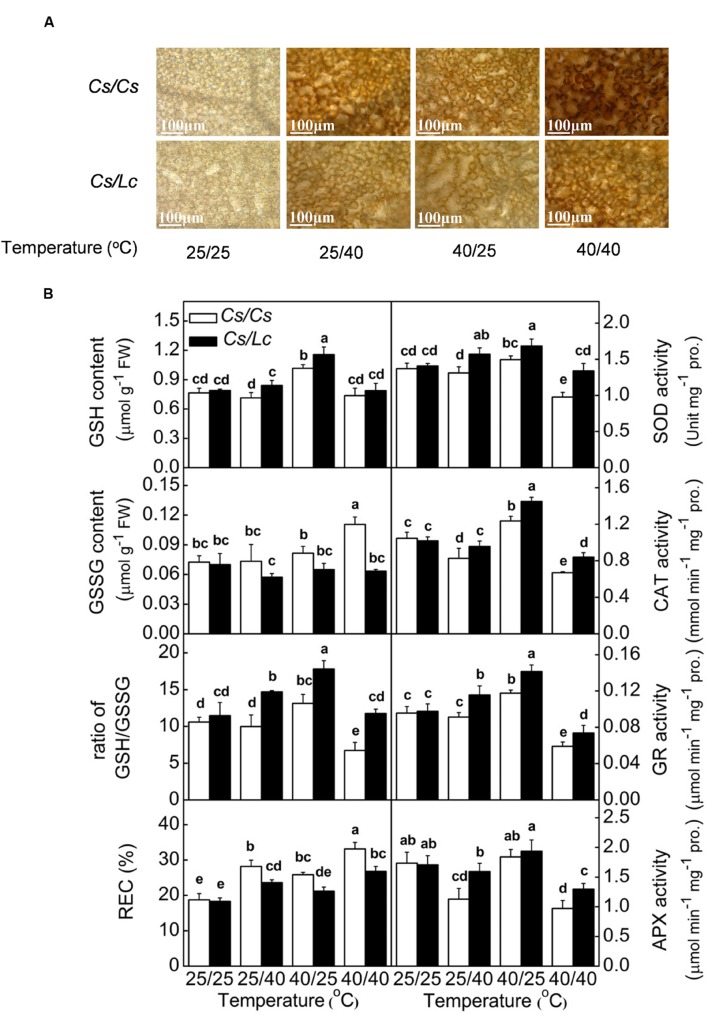
**Accumulation of H2O2 (A), antioxidant enzyme activity, glutathione homeostasis and membrane permeability reflected by relative electrical conductivity (REC) (B) in response to rootstock and root-zone or/and aerial heat stress.** Samples were harvested at 48 h after heat treatment. The bars (means ± SD, *n* = 4) labeled with different letters are significantly different at *P* < 0.05 according to Tukey’s test.

Inhibition of photosynthesis could induce generation of ROS such as superoxide (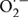) and hydrogen peroxide (H_2_O_2_). After *in situ* leaf staining with NBT and 3,3′-diaminobenzidine(DAB), we found intensive accumulation of 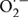 and H_2_O_2_ in *Cs/Cs* plants following heat treatments, where highest ROS accumulation was observed under combined heat treatment followed by root-zone heat and aerial heat (**Figure 5A**, **Supplementary Figure S1**). However, *Lc* as rootstock significantly reduced heat-induced accumulation of 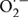 and H_2_O_2_. Similarly, relative electrical conductivity (REC) which reflected damage of cell membrane was obviously increased by heat-induced ROS in *Cs/Cs* plants, and this increase was attenuated by *Lc* rootstock.

Through evolution, plants have developed a set of antioxidant systems to remove excessive ROS that are generated by environmental stresses. The activities of major antioxidant enzymes such as SOD, CAT, GR, and APX in *Cs/Cs* plants were significantly decreased by overall heat stress. Only CAT and APX activities were obviously decreased by root-zone heat, while CAT and GR activities were increased by aerial heat treatment (**Figure 5B**). However, luffa rootstock alleviated the decreases or improved the increases of most enzyme activities by heat stress. As a result, the activities of most of these enzymes in *Cs/Lc* plants were significantly higher than those in *Cs/Cs* plants after different heat treatments. The content of GSH in *Cs/Cs* plants was increased by aerial heat but unchanged by root-zone and overall heat, while the GSSG content was increased by overall heat but unchanged by root-zone or aerial heat. Finally, the ratio of GSH/GSSG in *Cs/Cs* plants was increased by aerial heat, decreased by overall heat, but unchanged by oot-zone heat. With few exception, the contents GSH and GSSG in *Cs/Lc* plants were significantly higher and lower, respectively than those in *Cs/Cs* plants, after exposure to root-zone or/and aerial heat treatment. Thus, *Cs/Lc* plants had higher ratio of GSH/GSSG than *Cs/Cs* plants after heat treatments.

### The Role of HSP70 in *Lc*-Induced Protection of Photosynthesis in Cucumber Scion

**FIGURE 6:**
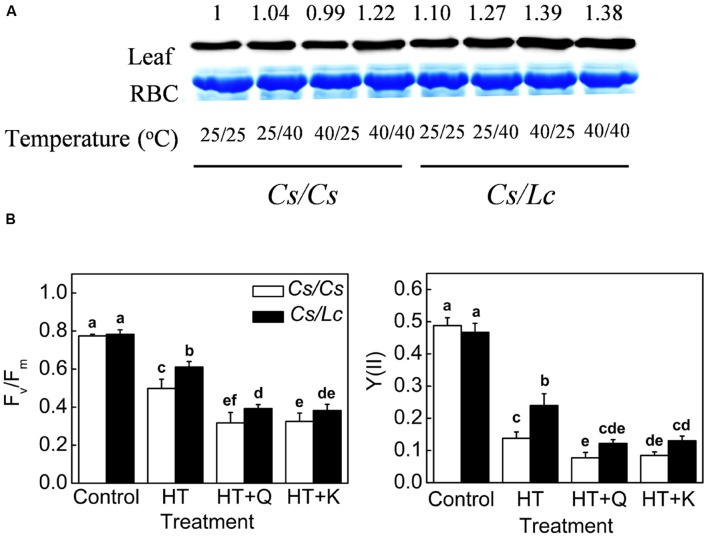
**HSP70 accumulation and its requirement in luffa rootstock-promoted photosynthetic improvement under heat stress.** **(A)** Changes in the level of HSP70 protein as influenced by rootstock and root-zone or/and aerial heat stress. Leaf samples were harvested at 48 h after heat treatments and analyzed by western blot. Protein was stained with Coomassie Brilliant Blue as a loading control. The same results were obtained in three independent experiments. **(B)** Changes in *F*_v_/*F*_m_ and the quantum efficiency of PSII (ΦPSII) in cucumber plants with cucumber or luffa roots as rootstock. Plants were pretreated with 200 μM KNK437 (K) or 200 μM Quercetin (Q) for 12 h and then plants were exposed to heat stress at 40/40°C (HT) for 48 h. *F*_v_/*F*_m_ and ΦPSII were measured at 48 h after heat stress. The bars (means ± SD, *n* = 4) labeled with different letters are significantly different at *P* < 0.05 according to Tukey’s test.

HSP70, an important molecular chaperone in heat shock proteins family, plays important roles in protecting proteins from heat-induced damage (Boston et al., 1996). HSP70 protein accumulation in *Cs/Cs* plants was almost unchanged by 25/40°C and 40/25°C, but was increased by 40/40°C. HSP70 in *Cs/Lc* plants was increased by all heat treatments to a higher level, compared to that in *Cs/Cs* plants (**Figure 6A**). Finally, the levels of HSP70 in *Cs/Lc* plants were increased by 1.27, 1.39, and 1.38 fold compared with those in *Cs/Cs* control, after 25/40°C, 40/25°C, and 40/40°C heat treatment for 48h, respectively. However, pretreatment with Q and K which are inhibitors of HSP70 expression, aggravated heat-induced damages as evident by drastic reduction in the *F*_v_/*F*_m_ and PSII in *Cs/Cs* and *Cs/Lc*, indicating that inhibition of HSP70 reduced the tolerance of grafted plants to heat stress and thus HSP70 might play a crucial role in *Lc* rootstock-mediated heat stress tolerance (**Figure 6B**).

## Discussion

Photosynthesis, an important process for energy production in plants, is very sensitive to various environmental stresses such as cold, heat, drought, heavy metal and so on (Takahashi and Murata, 2008). Grafting with stress-tolerant rootstocks can alleviate stress-induced reduction in photosynthesis. For example, grafting cucumber onto figleaf gourd and luffa alleviates chill- and heat-induced decrease in photosynthetic rate (Pn) by increasing Rubisco activity and decreasing oxidative stress (Zhou et al., 2007; Li et al., 2014b). When tomato is grafted onto heat-tolerant rootstock, sensitivity of photosynthesis to high temperature is decreased (Schwarz et al., 2010). In line with our previous study (Li et al., 2014a), the *A_sat_* and photosynthetic performance (PI_ABS_) in *Cs/Cs* plants were significantly decreased by root-zone heat (25/40°C), aerial heat (40/25°C), and combined heat (40/40°C) treatments. However, use of luffa, a heat-tolerant species, as rootstock alleviated such photosynthetic inhibition (**Figure 1**). Consistently, grafted tomato plants with heat tolerant tomato or eggplant cultivar as rootsstock showed higher photosynthetic performance based on the higher values of chlorophyll fluorescence under heat stress conditions (Abdelmageed and Gruda, 2009). These observations further confirms that grafting is an efficient strategy for improving heat tolerance in horticultural crops.

Photon flux is absorbed by the antenna pigments that excite chlorophyll, and part of the excitation energy (the other is dissipated as heat and fluorescence emission) is converted to redox energy via electron transport and leads to ultimate CO_2_ fixation (Strasser and Strasser, 1995). A number of studies have suggested that electron transport is highly heat sensitive and it is the main functional limitation to photosynthesis at high temperature (Wise et al., 2004; Hüve et al., 2006). Both photosystem (PS) II and I behavior at high temperature has been extensively studied; consistently, PSII was demonstrated more likely to be injured by high temperature than PSI (Sayed et al., 1989; Mihailova et al., 2011; Essemine et al., 2012; Yan et al., 2013). In the present study, both electron transport driven by PS II and I were markedly restricted by root-zone heat and aerial heat, especially by the former one. And consistently, greater decrease in PS(II) and ETR(II) suggested that PSII was more susceptible to heat stress than PSI. Higher tolerance of PSI to heat was contributed to its higher light protection capacity [Y(ND)] to some extent (**Table 1**).

Restriction of electron transport in PS II after aerial heat stress perhaps attributed to direct injury to the photosynthetic apparatus. PSII donor side OEC is often recognized as the most heat-sensitive PSII component (Allakhverdiev et al., 2008; Li et al., 2009). Inactivation of OEC by heat stress results in structural changes of D1 and D2 protein and then affects Q_A_ fixation and stability of PSII (Sinsawat et al., 2004; Camejo et al., 2005). However, compared with OEC, changes in φ_E0_ and qP were more sensitive to aerial heat stress as shown in our results (**Table 1**; **Figure 2**), and these indicated severe depression in electron transport beyond Q_A_. Thus, other than PSII donor side, acceptor side maybe the primary limitation site of photosynthesis system by aerial heat stress. The underlying reason is that aerial heat stress was carried out under growth light not in dark, and the block of electron transport from PSII may be a self-protection way by reducing the possibility of PSI photoinhibition (Sonoike, 2011). In addition, root-zone heat almost unaffected OEC activity, but inhibited the activity of acceptor side such as Q_A_ and PQ, suggesting acceptor side of PSII was more sensitive to heat. More importantly, *Lc* rootstock alleviated root-zone heat-caused restriction of acceptor side and aerial heat-caused restriction in donor and acceptor sides in cucumber shoot.

At the end of linear electron transport chain, electron is transported to NADP^+^ to generate NADPH which can be consumed in CO_2_ fixation that regenerates NADP^+^ again (Farquhar et al., 1980). The ratio of NADP^+^/NADPH was increased by aerial heat which inhibited electron transport in PSII and then reduced the rate of NADPH generation. However, the ratio of NADP^+^/NADPH was decreased by root-zone heat (**Figure 3**), which inhibited Calvin cycle capacity by depressing Rubisco (a key enzyme involved in Calvin cycle) activation state and activity and then reduced the rate of NADP^+^ regeneration. It has been well documented that activation state of Rubisco is a crucial limiting factor for photosynthesis, and Rubisco activase is highly susceptible to high temperature (Salvucci and Crafts-Brandner, 2004; Scafaro et al., 2012). Rubisco activation state and activity are closely related with supply of substrate CO_2_ concentration in chloroplast. For instance, water stress inhibits Rubisco activity significantly by decreasing *Gs* (Flexas et al., 2006; Galmés et al., 2011). Likewise, root-zone heat stress decreased the leaf water potential and *Gs* in *Cs/Cs* plants by reducing root vitality, prevented the entry of CO_2_, and then resulted in decreased Rubisco activity (**Figures 4** and **5**). It is worth mentioning that limited supply of CO_2_ accelerates photodamage to PSII via the excessive reduction of Q_A_ (Melis, 1999; **Figure 2**), inhibits the repair of photodamaged PSII (Takahashi and Murata, 2005) and synthesis of D1 protein in intact chloroplasts (Takahashi and Murata, 2006). Down regulation of PSII activity results in imbalance between the generation and utilization of electrons and photoinhibition. To dissipate excess light energy, excessive electron is transported to molecular oxygen, a competitor with NADP^+^, thus generating active oxygen species (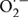, ^1^O_2_, H_2_O_2_, ⋅OH), which are potentially dangerous for plants (**Figure 5**). Excessive ROS block the electron transport by affecting the repair process of PSII and inducing cleavage aggregation of RC proteins, which eventually form a vicious cycle (Allakhverdiev et al., 2008). Moreover, ROS could move to thylakoid and cell membranes, thus causing increased membrane lipid peroxidation, reduced membrane stability, and increased membrane permeability (Sharma et al., 2012). We found heat stress induced accumulation of H_2_O_2_ and 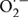 in *Cs/Cs* and *Cs/Lc* plants (**Figure 5A**; **Supplementary Figure S1**), and the increase of REC indicated excess ROS caused membrane damage (**Figure 5B**). The antioxidant system including antioxidant enzymes and non-enzymatic antioxidants is responsible to remove excessive ROS under moderate stress and suppress cell death under severe stress (Ahmad et al., 2010). Previously, we have shown that grafting onto tolerant rootstock could alleviate stress-induced oxidative stress by enhancing antioxidant system (Li et al., 2014b). Likewise, *Lc* rootstock improved antioxidant enzyme activity and increased ratio of GSH/GSSG and thus minimizing ROS accumulation under heat stress.

Furthermore, stress-tolerant rootstock could trigger gene transcription and the expression of proteins involved in stress responses such as HSP70 in the scion via some long-distance signal(s) (Li et al., 2014a). As a typical molecular chaperone, apart from protecting antioxidant enzymes from denaturation or inactivation (Boston et al., 1996; Hu et al., 2010; Li et al., 2014b), HSP70 also plays a role in the photoprotection and repair of PSII during and after photoinhibition often caused by various stresses including heat (Murata et al., 2007; Takahashi and Murata, 2008). HSP70 protein was significantly induced by *Lc* rootstock at earlier stage 12 h of heat treatments (Li et al., 2014a), and this induction was retained until later stage (48 h) (**Figure 6**). According to our previous results, the induction of HSP70 by luffa rootstock was closely related to luffa rootstock-induced increase of ABA content (Li et al., 2014a). Quercetin and KNK437 (*N*-formy1-3,4-methylenedioxy-benzy-lidene-g-butyrolactam) are inhibitors of HSP70 synthesis in plant and animal cells (Manwell and Heikkila, 2007; Hu et al., 2010). More importantly, inhibition of HSP70 by Quercetin or KNK437 attenuated *Lc* rootstock- induced heat tolerance in PSII of *Cs/Lc* plants. Therefore, HSP70 consistently played an important role in *Lc* rootstock that maintained PSII activity and reduced oxidative stress under heat stress.

## Conclusion

Root-zone heat stress inhibited photosynthesis mainly through decreasing Rubisco activity, while aerial heat stress mainly through inhibiting PSII acceptor side in cucumber plants. Restriction of electron transport resulted in accumulation of ROS which caused damage to photosynthetic apparatus, forming a vicious cycle. In the field condition, aerial temperature fluctuates frequently, and thus PSII acceptor side is to be considered as the primary limiting site of photosynthesis under heat stress. Grafting onto *Lc* rootstock alleviated heat-induced photosynthetic inhibition by maintaining higher root vitality and by inducing accumulation of HSP70. HSP70 was potentially involved in photoprotection and repair of PSII, as well as activation of antioxidant enzymes.

## Author Contributions

HL and YZ designed research; HL, GA, and GZ performed research; HL, XX, JZ, KS, JY, and YZ analyzed data; HL, GJ, and YZ wrote and revised the paper.

## Conflict of Interest Statement

The authors declare that the research was conducted in the absence of any commercial or financial relationships that could be construed as a potential conflict of interest.

The reviewer AS-S and handling Editor declared their shared affiliation, and the handling Editor states that the process nevertheless met the standards of a fair and objective review.

## Abbreviations

*A_sat_*: light saturated photosynthetic rate
ABS/CS_m_: photon flux absorbed by the antenna pigments per cross section
APX: ascorbate peroxidase
CAT: catalase
Chl(a+b): chlorophyll a and b
*Ci*: intercellular CO_2_
*F*_v_/*F*_m_: the maximum photochemical efficiency of PSII
GR: glutathione reductase
*Gs*: stomatal conductance
GSH: reduced glutathione
GSSG: oxidized glutathione
HSP70: heat shock protein 70
K: KNK437
MDA: malondialdehye
OEC: oxygen-evolving complex
PI_ABS_: photosynthetic performance index
PPDF: photosynthetic photon flux density
Q: Quercetin
qP: a parameter estimating the fraction of open PS II centers based on apuddle model
ROS: reactive oxygen species
SOD: superoxide dismutase
Y(I): effective quantum yield of PSI
Y(II): effective quantum yield of PSII
φ_E0_: quantum yield of electron transport beyond Q_A_
φ_P0_: the maximum quantum yield of primary photochemistry
