# Supplementary material for: Unraveling Main Limiting Sites of Photosynthesis under Below- and Above-Ground Heat Stress in Cucumber and the Alleviatory Role of Luffa Rootstock
Source: Front Plant Sci. 2016 Jun 2;7:746. doi: 10.3389/fpls.2016.00746 (PMC4889590; doi:10.3389/fpls.2016.00746)
Supplement: Supplementary file 1 [file Data_Sheet_1.DOC]

**Supplementary Figure 1.** Changes in the accumulation of (A) O_2_^.-^ and (B) H_2_O_2_ in cucumber plants as influenced by rootstock and root-zone or/and aerial heat stress.

**
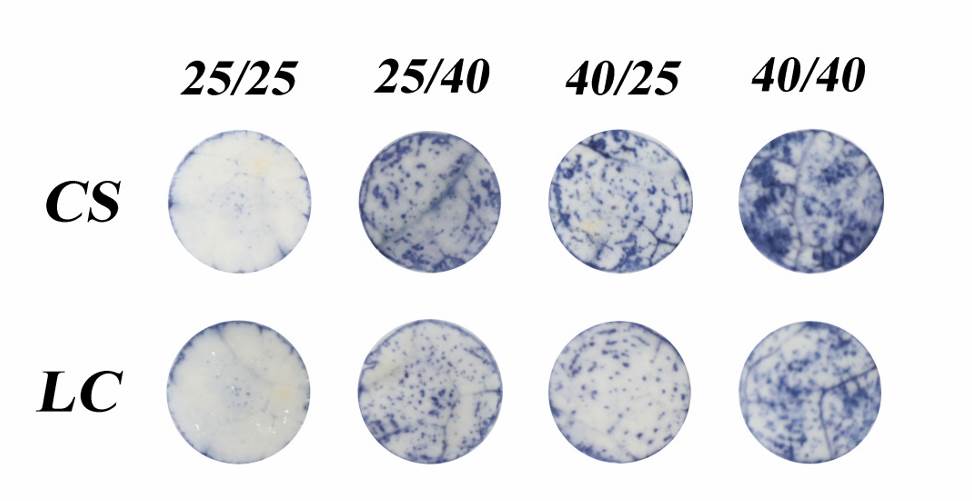
**

25/25

40/25

40/40

25/40


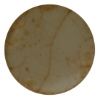

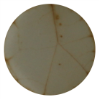

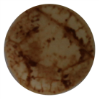

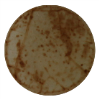

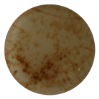

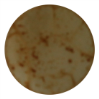

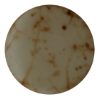

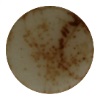


25/40

40/40

40/25

25/25

*Cs/Cs*

*Cs/Lc*

Temperature (^o^C)

(A)

(B)

**Supplementary Table 1.** The formulae and illustrations of JIP-test parameters.

| Formulae and terms | Illustrations |
| --- | --- |
| V_K_=(F_300μs_-F_0_)/( F_m_-F_0_)  V_j_=(F_j_-F_0_)/(F_m_-F_0_)  M_0_=4(F_300μs_-F_0_)/(F_m_-F_0_)  S_m_=(Area)/ (F_m_-F_0_)  φ_P0_=F_V_/F_m_=[1-(F_0_/F_m_)]  φ_E0_=[1-(F_0_/F_m_)]ψ_0_  ψ_0_=(1-V_J_)  ABS/CS_m_≈F_m_  TR/CS_M_=φ_P0_ (ABS/CS_m_)  ET/CS_m_=φ_E0_ (ABS/CS_m_)  OEC=[1-(V_K_/V_J_)_treated_]/[1-(V_K_/V_J_)_control_]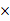100  RC/CS_m_=φ_P0_ (V_J_/M_0_) (ABS/CS_m_)  PI_ABS_=(RC/ABS)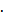[φ_P0_/(1-φ_P0_)] ∙[ψ_0_/(1-ψ_0_)] | Relative variable fluorescence at step K  Relative variable fluorescence intensity at the J-step  Approximated initial slope of the fluorescence transient  Normalized total complementary area above the O-J-I-P transient (reflecting multiple turnover Q_A_ reduction events)  Maximum quantum yield of primary photochemistry (at t=0)  Quantum yield of electron transport (at t=0)  Probability that a trapped exciton moves an electron into the electron transport chain beyond Q_A_^-^ (at t=0)  Absorption flux per CS, approximated by F_M_  Trapped energy flux per CS  Electron transport flux per CS  Oxygen evolving complex  Density of RCs (QA-reducing PSII reaction centers)  Performance index on absorption basis |
